# Anticoagulation for Cancer Patients in Special Situations: A Narrative Review of Guidelines and Literature

**DOI:** 10.3390/cancers18111707

**Published:** 2026-05-23

**Authors:** Pilar Sotoca Rubio, Juan José Serrano Domingo, Patricia Guerrero Serrano, Patricia Pérez de Aguado Rodríguez, Ana María Barrill Corpa, Jaime Moreno Doval, Coral García de Quevedo Suero, Juan Carlos Calvo Pérez, Carlos González-Merino, Guillermo González Martín, Jesús Chamorro Pérez, Ana Gómez Rueda, Pilar Garrido López

**Affiliations:** 1 Department of Medical Oncology, Ramon y Cajal University Hospital, 28034 Madrid, Spain; patricia.guerrero@salud.madrid.org (P.G.S.); patricia.perezaguado@salud.madrid.org (P.P.d.A.R.); jmdoval@salud.madrid.org (J.M.D.); cgquevedosuero@salud.madrid.org (C.G.d.Q.S.); jcalvop@salud.madrid.org (J.C.C.P.); cgmerino@salud.madrid.org (C.G.-M.); guillermo.gonzalez.martin@salud.madrid.org (G.G.M.); jchamorro@salud.madrid.org (J.C.P.); pgarrido@salud.madrid.org (P.G.L.); 2 Department of Medical Oncology, Vithas La Milagrosa University Hospital, 28010 Madrid, Spain; 3 Department of Medical Oncology, Infanta Leonor University Hospital, 28031 Madrid, Spain; anamaria.barrill@salud.madrid.org; 4 Department of Medical Oncology, Príncipe de Asturias University Hospital, 28805 Alcalá de Henares, Spain; agomezrueda@salud.madrid.org

**Keywords:** cancer-associated thrombosis, venous thromboembolism, low-molecular-weight heparin, direct oral anticoagulant, recurrent thrombosis, thrombocytopenia, renal impairment, central venous catheter-associated thrombosis, atypical site thrombosis, clinical practice guidelines

## Abstract

Cancer-associated thrombosis (CAT) is a major cause of morbidity and mortality in patients with cancer. The management of special situations—including recurrent venous thromboembolism (VTE), thrombosis at unusual sites, and central venous catheter-associated thrombosis (CVC-AT)—remains particularly challenging because of the limited availability of high-quality evidence. This narrative review synthesizes recommendations from major international and Spanish clinical practice guidelines and expert consensus documents, including those from SEOM, ESMO, ASCO, NCCN, ITAC and SEMI, to provide a structured framework for the management of these complex scenarios. Our analysis identified substantial heterogeneity across guidelines, particularly regarding anticoagulant selection, dosing strategies, and treatment duration. Although some convergence exists in the management of CVC-AT, important discrepancies and evidence gaps persist in areas such as splanchnic vein thrombosis, hepatic impairment, central nervous system involvement, and recurrent VTE despite treatment. In many cases, recommendations are based primarily on expert opinion rather than robust trial data, and several clinical scenarios are addressed by only a limited number of guidelines. These findings underscore the need for more standardized management strategies and prospective clinical studies to better inform decision-making in daily practice. Overall, this review highlights the growing importance of individualized anticoagulant management aimed at balancing thrombotic and bleeding risks in high-risk oncology patients, thereby helping to bridge the gap between expert consensus and evidence-based precision anticoagulation.

## Simple Summary

Cancer-associated thrombosis is a frequent and potentially life-threatening complication. Although general treatment strategies are well established, significant uncertainties remain in special situations. This review aims to synthesize current international guidelines and expert consensus to provide clear and practical management strategies for these challenging situations. By highlighting areas of diagnostic uncertainty and the limited evidence derived from clinical trials, this work provides a clinically oriented framework to support individualized therapeutic decision-making. Ultimately, this review seeks to promote a more standardized approach to care in underrepresented clinical settings, with the potential to improve safety outcomes and survival in oncology patients at high thrombotic risk.

## 1. Introduction

Cancer-associated thrombosis (CAT) is one of the most important complications in oncology patients, and represents the second leading preventable cause of death in this population after cancer progression [1,2,3]. This condition most commonly manifests as venous thromboembolism (VTE), including deep vein thrombosis (DVT) and pulmonary embolism (PE), although thrombosis may also occur in atypical sites such as the splanchnic circulation or central nervous system (CNS) [2,3].

The incidence of VTE in cancer patients is approximately ninefold higher than in the general population and may be even greater in specific high-risk subgroups. The introduction of novel anticancer therapies, population aging, and the increased use of central venous catheters (CVCs) have all contributed to this rising incidence [2,4]. In addition, approximately 20% of VTE episodes occur in patients with active malignancy [1].

Multiple factors contribute to the pathogenesis of CAT, including tumor-related characteristics (histology, stage, anatomical location), treatment-related factors (chemotherapy, surgery, targeted therapies, immunotherapy), and patient-related factors (comorbidities, immobility, personal or family history of thrombosis, thrombophilia). Certain malignancies, particularly pancreatic, gastric, lung, brain tumors, and hematologic malignancies, are associated with a particularly high thrombotic risk [2,5,6].

The clinical management of VTE in cancer patients remains especially challenging because of the delicate balance between the risk of recurrent thrombosis and anticoagulant-associated bleeding complications [6]. Compared with non-cancer patients, individuals with cancer have recurrence rates up to three times higher despite adequate anticoagulation, while the risk of major bleeding may be up to twofold higher, especially in the presence of metastases, gastrointestinal involvement, or chemotherapy-induced thrombocytopenia [3,7].

Thrombosis may negatively affect the prognosis of cancer patients, not only because of its direct contribution to mortality, but also through delays or interruptions in anticancer therapy and its impact on quality of life. The occurrence of VTE at cancer diagnosis or during the first year thereafter has been associated with worse overall oncologic outcomes [2,4].

In this context, early identification of patients at risk of CAT, appropriate thromboprophylaxis in selected patients, and individualized anticoagulant therapy are essential components of modern oncology care. Predictive models such as the Khorana score and its derivatives (PROTECHT, ONKOTEV, COMPASS) can support risk stratification, although their use in routine clinical practice remains limited [1,3,5,8,9]. In this setting, the role of anticoagulation in the treatment of VTE and in both inpatient and outpatient prophylaxis is well established.

However, certain clinical scenarios represent special situations in which VTE management and anticoagulant treatment remain particularly challenging. This is usually due to limited evidence available for these “special situations” which makes it harder for clinical decision-making [1,5,8,9].

## 2. Methodology

Given the limited level of evidence available for most of the clinical scenarios addressed in this review, a comprehensive evaluation of the main clinical practice guidelines and selected expert consensus statements focused on oncology patients was performed. Both international and Spanish sources were included to synthesize and compare their respective recommendations. In several instances, guidelines provide concordant recommendations; however, discrepancies remain in other areas. Moreover, not all guidelines address the same clinical scenarios. In situations in which consensus is lacking or recommendations are unclear or unavailable, the authors’ perspectives and proposed management strategies are presented.

The guidelines and consensus documents evaluated include:

- Spanish Society of Medical Oncology (SEOM, for its acronym in Spanish), 2023: “SEOM Clinical Guidelines on Venous Thromboembolism and Cancer” [1];
- European Society for Medical Oncology (ESMO), 2023: “Venous thromboembolism in cancer patients: ESMO Clinical Practice Guideline” [2];
- American Society of Clinical Oncology (ASCO), 2020: “Venous thromboembolism prophylaxis and treatment in patients with cancer: ASCO clinical practice guideline update” [8], and the 2023 update [9];
- National Comprehensive Cancer Network (NCCN): “Cancer-Associated Venous Thromboembolic Disease, Version 3.2025” [4];
- International Initiative on Thrombosis and Cancer (ITAC): “2022 International Clinical Practice Guidelines for the Treatment and Prophylaxis of Venous Thromboembolism in Patients with Cancer, including Patients with COVID-19” [3];
- Spanish Society of Internal Medicine (SEMI, for its acronym in Spanish): “2024 Recommendations for the Management of Cancer-Associated Venous Thrombosis” [6];
- SEMI, SEOM and Spanish Society of Thrombosis and Hemostasis (SETH): “Cancer-associated thrombosis: beyond clinical practice guidelines—A multidisciplinary expert consensus” [5];
- “Consensus on the prevention and treatment of cancer-associated thrombosis in controversial clinical scenarios with low levels of evidence” [10].

References were also made to multiple randomized clinical trials and observational studies evaluating the use of anticoagulants in both cancer and non-cancer populations. For the management of patients with renal or hepatic impairment, recommendations were based on the summaries of product characteristics (SmPCs) issued by the European Medicines Agency.

In addition, several targeted literature searches were conducted to further support and contextualize specific recommendations. These searches were performed in the PubMed database and focused on anticoagulation in patients with CNS involvement, the management of anticoagulation in patients with extreme body weights, and the use of unfractionated heparin (UFH) in patients with cirrhosis.

## 3. Special Situations

### 3.1. Thrombotic Recurrence During Anticoagulant Therapy

Thrombotic recurrence is defined as either progression of a previously diagnosed VTE or the occurrence of a new VTE event while the patient is receiving therapeutic-dose anticoagulation [4]. The reported incidence of recurrent thrombosis in patients receiving anticoagulant therapy ranges from 4% to 11% [1]. Major risk factors include advanced tumor stage, metastatic disease, chemotherapy or other thrombogenic therapies, locally advanced tumors causing vascular compression, tumor histology, disease progression, molecular tumor characteristics, and a prior history of VTE. To date, no validated predictive models are available to estimate recurrence risk, and no randomized controlled trials (RCTs) have specifically addressed therapeutic decision-making in this setting [1]. Consequently, current recommendations are largely based on expert consensus and require individualized clinical assessment.

The initial approach should focus on identifying potential causes of therapeutic failure:

- Verification of appropriate anticoagulant use, including assessment of treatment adherence and potential administration errors [1,4,6,8,10].
  ○ This may require laboratory monitoring, including INR for vitamin K antagonists (VKA), activated partial thromboplastin time (aPTT) for UFH, or anti-Xa levels for UFH and low-molecular-weight heparin (LMWH) [4].
- Evaluation of potential drug–drug and drug–food interactions [1,4].
- Exclusion of heparin-induced thrombocytopenia (HIT) [1,4,6,8,10].
- Assessment of tumor progression [1,6,10].
- Identification of anatomical compressions (vascular or lymphatic) [4,8]. Correcting these factors can be essential to reduce the risk of recurrence [4].

When defining the therapeutic strategy after thrombotic recurrence, several factors should be considered, including the anticoagulant currently used, evidence of tumor progression, and the presence of additional transient risk factors that may have triggered a new episode of VTE. Bleeding risk, the reasons for dose reduction (if applicable), and whether the thrombosis represents a new event or progression of a prior thrombosis should also be considered [1].

Based on these considerations, the following clinical scenarios and management strategies are proposed:

- Patients receiving UFH: Therapeutic aPTT and anti-Xa levels should be confirmed. In cases of subtherapeutic levels, dose escalation is recommended. If therapeutic levels are documented, switching to an alternative anticoagulant (LMWH, direct oral anticoagulant [DOAC], or fondaparinux) should be considered [4].
- Patients receiving subtherapeutic LMWH doses: Escalation to therapeutic dosing or switching to a DOAC [1,4], fondaparinux, or UFH [4].
- Patients receiving therapeutic LMWH doses: Dose escalation by approximately 25% [1,3,4,5,6], transition to a twice-daily LMWH regimen [1,4], or switching to a DOAC [1,3,4,6,10], fondaparinux, or UFH [4]. Among these options, LMWH dose escalation is supported by evidence from two retrospective studies [1]. Although clinical guidelines recommend a 25% increase in LMWH dose, the literature suggests that, in routine clinical practice, dose escalation typically ranges from 20% to 33% [11].
- Patients receiving DOAC therapy: Switching to LMWH [1,3,4,6,10] fondaparinux, or UFH [4].
- Patients receiving fondaparinux: Switching to LMWH, a DOAC, or UFH [4].
- Patients receiving VKA: Switching to LMWH, a DOAC [1,3,4,6,10], fondaparinux, or UFH [4].

Recommendations to switch to fondaparinux or UFH are explicitly included only in the NCCN guidelines [4]. Whenever feasible, UFH should be reserved for the inpatient setting, and fondaparinux should primarily be considered when other anticoagulant options are contraindicated or not feasible.

The 2020 ASCO guidelines [8] provide limited guidance in this scenario, recommending either switching to an alternative anticoagulant or escalating the LMWH dose without specifying the extent of dose increase. Based on recommendations from other guidelines, this approach may include switching from a DOAC to LMWH or vice versa, with fondaparinux remaining a potential option in selected cases. Certain DOACs were not recommended in the 2020 ASCO guidelines; these limitations were addressed in the 2023 update [9].

Finally, some guidelines consider inferior vena cava filter (IVCF) placement following thrombotic recurrence, although only as a last-resort intervention in patients with recurrent events despite optimized anticoagulation, given the lack of demonstrated survival benefit and the increased long-term risk of DVT [3,8,10]. The ITAC guidelines restrict this indication to cases of recurrent PE [3]. Anticoagulation should be continued despite IVCF placement.

### 3.2. Central Venous Catheter-Associated Thrombosis

The reported incidence of central venous catheter-associated thrombosis (CVC-AT) varies widely, ranging from 2% to 66% in asymptomatic patients and from 2.7% to 13.8% in symptomatic patients. Incidence also varies according to the type of device used and appears to be lower with totally implantable ports than with peripherally inserted central catheters (PICCs) (2% vs. 11%; OR 0.20) [1,2].

Despite the availability of multiple clinical practice guidelines addressing CVC-AT, substantial heterogeneity persists among recommendations. Several clinically relevant issues remain unresolved or are insufficiently addressed, complicating clinical decision-making. These discrepancies likely reflect differences in publication dates, as well as regional variations in available resources and therapeutic strategies. In this context, a recent review identified areas of agreement, divergence, and evidence gaps across published guidelines for the management of CVC-AT, focusing on clinically relevant priorities [12]. This review included not only oncology-focused guidelines, but also recommendations from other medical specialties, encompassing a total of 16 scientific societies: European Society of Cardiology (ESC) [13], European Society for Vascular Surgery (ESVS) [14], American College of Chest Physicians (CHEST) [15], British Society for Haematology (BSH) [16], Società Italiana di Nutrizione Clinica e Metabolismo (SINuC) [17], European Society for Clinical Nutrition and Metabolism (ESPEN) [18], ESMO [2], American Society of Hematology (ASH) [19,20], NCCN [21], ITAC [3], American Society of Clinical Oncology (ASCO): “Central Venous Catheter Care for the Patient with Cancer” [22], International Society on Thrombosis and Haemostasis (ISTH) [23,24], SEOM [25], French National Federation of Cancer Centers (SOR) [26], Association of Anaesthetists of Great Britain and Ireland (AAGBI) [27] and Scandinavian Society of Anaesthesiology and Intensive Care Medicine (AAS) [28].

Therefore, the recommendations summarized for CVC-AT will include not only those from the main oncology-focused guidelines [1,2,3,4,5,6,8,9], but also the conclusions of the aforementioned review [12]. In addition, ASCO guidelines has published a specific document addressing this clinical scenario [22], which was also included in the review by Rosson et al. [12]. The SEOM and NCCN guidelines cited by Rosson et al. correspond to earlier versions than those included in our review. In general, recommendations on this topic have remained consistent across guideline updates; however, any relevant changes have been specifically highlighted when applicable.

#### 3.2.1. Thromboprophylaxis in CVC-AT

A Cochrane systematic review concluded that prophylactic administration of LMWH reduces the incidence of CVC-AT without increasing bleeding risk (RR 0.43) [29]. In addition, a prospective non-randomized study in patients with CVCs demonstrated lower rates of CVC-AT among patients receiving prophylaxis with rivaroxaban or LMWH compared with no prophylaxis (3.76% with rivaroxaban, 3.03% with LMWH, and 12.4% without prophylaxis) [30].

Nevertheless, most clinical guidelines do not recommend routine thromboprophylaxis in patients with CVCs [1,2,3]. However, the ASH and AAGBI guidelines suggest that prophylaxis may be considered in selected high-risk patients: specifically, ASH recommends considering prophylaxis in patients receiving agents such as thalidomide, lenalidomide, or pomalidomide (commonly used in multiple myeloma) [19,20], whereas AAGBI suggest prophylaxis in patients with a prior history of VTE [27]. In addition, the SEMI–SEOM–SETH consensus (not included in the Rosson et al. review) suggests prophylactic-dose LMWH for at least 30 days in patients with a prior history of CVC-AT requiring placement of a new CVC, with consideration of extending prophylaxis for as long as the catheter remains in place [5].

Therefore, although routine prophylaxis for CVC-AT is generally discouraged, we believe that the recommendations proposed by the SEMI–SEOM–SETH consensus should be taken into consideration. Accordingly, thromboprophylaxis may be considered in selected patients with a history of CVC-AT requiring insertion of a new CVC.

#### 3.2.2. Strategies to Reduce the Risk of CVC-AT

Most clinical guidelines recommend right-sided catheter placement (particularly for internal jugular vein access), with the catheter tip positioned at the junction between superior vena cava and the right atrium [1,2,3,4]. Rosson et al.’s review reported that the ISTH, AAGBI and ESPEN guidelines are consistent with this recommendation [12]. In contrast, the ASCO guidelines consider both right- and left-sided placement acceptable depending on patient characteristics, arguing that the available evidence is insufficient to support a specific recommendation [22]. Although the ESPEN guidelines recommend right-sided placement, they also acknowledge that the supporting evidence is limited [18].

Additional recommendations, particularly regarding the use of small-caliber catheters, are provided by ESPEN, ASCO, AAGBI, and SINuC [17,18,22,27]. Specifically, SINuC guidelines recommend that catheter diameter should not exceed 33% of the vein diameter [17]. When specified (ESPEN, ASCO), femoral catheter placement is discouraged [18,22].

In summary, the review by Rosson et al. concludes that the available evidence supports the use of small-caliber CVCs inserted under ultrasound guidance through the internal jugular vein, with the catheter tip positioned at the junction of superior vena cava and the right atrium. Femoral access should be avoided whenever possible, and thoracic or implantable ports should be preferred over femoral or peripherally inserted catheters. Appropriate nursing care and prompt catheter removal when no longer indicated are also emphasized [12].

#### 3.2.3. Management of Established CVC-AT

Once CVC-AT has been diagnosed, catheter removal should not be systematic, and catheter preservation should be attempted whenever feasible [1,2,3,4,5,6,8,9,12,22]. The main indications for CVC removal cited across guidelines include:

- Contraindications to anticoagulation [1,2,4];
- Catheter-related infection [1,2,3,4,5];
- Lack of ongoing need for the catheter [1,2,4] or catheter dysfunction, including malposition [3,4,5];
- Progression of CVC-AT despite anticoagulation associated with clinical deterioration [1,2,5];
- Situations in which thrombosis poses a threat to life or physical integrity, or when catheter reinsertion is considered technically straightforward [12].

Only the SINuC, ISTH, SOR, and SEOM guidelines recommend initiating anticoagulation before catheter removal [1,17,23,24,25,26]. The minimum recommended duration of anticoagulation prior to removal varies across guidelines, ranging from 3–5 days (ISTH) [23,24] and 5 days (SINuC) [17] to 5–7 days (SEOM) [1]. A duration of approximately 5 days appears reasonable unless catheter retention poses an immediate life-threatening risk. When anticoagulation is contraindicated, NCCN guidelines recommend immediate catheter removal or ultrasound surveillance until the contraindication resolves [4].

#### 3.2.4. Anticoagulant Therapy

All guidelines recommend anticoagulation for patients with CVC-AT for a minimum duration of 3 months [1,2,3,4,5,6,8,9,12,22], with some extending treatment to 3–6 months (SEOM, BSH, ASH, ASCO, ISTH) [1,16,19,20,22,23,24]. If the catheter is not removed, several guidelines recommend considering indefinite anticoagulation, particularly in patients with active cancer (SEOM, ESMO, ITAC, NCCN, SEMI, SEMI–SEOM–SETH, CHEST, SINuC) [1,2,3,4,5,6,15,17]. The SINuC guidelines and the SEMI–SEOM–SETH consensus suggest that, after the initial 3 months, treatment may be continued using prophylactic [5,17] or intermediate doses [5] of LMWH.

There is no clear consensus regarding the optimal anticoagulant agent, and recommendations include LMWH, DOACs and VKA, largely reflecting differences in publication dates among the various guidelines. Since several guidelines address CVC-AT beyond the oncology setting, recommendations regarding VKA are not emphasized here because they are generally not recommended in cancer patients. LMWH is recommended by most guidelines [1,2,3,5,6,14,16,18,22,23,24,26]. Only the ESC and ASH guidelines consider DOACs as first-line therapy [13,19,20], whereas SEOM and ESMO consider them alternative option [1,2].

In our opinion, CVC-AT should not be considered a distinct thrombotic entity but rather a subtype of DVT occurring in cancer patients. Consequently, anticoagulant selection should follow the same principles used for other forms of CAT, taking into account patient- and tumor-related factors, with LMWH or DOACs as first-line options. Fondaparinux or UFH may be considered in selected cases when these agents are contraindicated.

#### 3.2.5. Thrombolysis and Invasive Interventions

Thrombolysis or surgical thrombectomy may be considered in selected symptomatic patients who fail to improve despite anticoagulation, although supporting evidence remains limited. Thrombolysis is mentioned several guidelines [3,4,6,13,14,15,17,18,22,23,24,26,27,28], whereas ESPEN recommends its use primarily in acute symptomatic cases [18]. Important factors to consider before thrombolysis include:

- Presence of severe symptoms (ESC, ESVS, SINuC, ISTH) [13,14,17,23,24];
- Low bleeding risk (ITAC, SINuC, ISTH) [3,17,23,24];
- Intent to preserve the catheter (SINuC, ASCO, ISTH) [17,22,23,24].

Regarding invasive techniques (thrombectomy), the ESVS guidelines do not recommend early thrombus removal in most symptomatic patients [14]. The SINuC guidelines consider thrombectomy only in patients with persistent symptoms despite anticoagulation and thrombolysis, or when the goal is to improve venous patency, shorten anticoagulation duration, or reduce the risk of post-thrombotic syndrome, while acknowledging that evidence supporting these indications remains limited [17]. NCCN and SEMI guidelines also consider thrombectomy in selected scenarios, such as thrombotic superior vena cava syndrome or phlegmasia cerulea dolens [4,6].

#### 3.2.6. Summary of Recommendations

In summary:

- Routine thromboprophylaxis is not recommended in patients with CVCs. However, it should be considered in patients with a prior history of CVC-AT requiring placement of a new catheter, preferably using LMWH, although recent evidence also supports the use of DOACs (e.g., rivaroxaban).
- Most guidelines recommend the use of small-caliber CVCs inserted through the internal jugular vein, with the catheter tip positioned at the junction of the superior vena cava and the right atrium. Femoral access should be avoided whenever possible, and implantable ports should be preferred over PICCs. Catheters should be removed as soon as they are no longer clinically indicated.
- Following diagnosis of CVC-AT, catheter preservation should be attempted whenever feasible. Indications for catheter removal include contraindications to anticoagulation, catheter-related infection, catheter dysfunction or lack of ongoing clinical need, progression despite anticoagulation, or life-threatening thrombosis. Ease of reinsertion alone should not justify catheter removal.
- Anticoagulation should ideally be initiated before catheter removal, with a minimum duration of 3–7 days before removal (approximately 5 days appears reasonable), unless immediate removal is required for safety reasons.
- If anticoagulation is contraindicated, catheter removal or ultrasound surveillance should be considered.
- Regardless of whether the catheter is removed, anticoagulant therapy should be maintained for at least 3 months. Longer treatment duration should be considered in patients with active cancer and retained catheters. After 3 months, de-escalation to prophylactic or intermediate-dose LMWH may be considered.
- Anticoagulant selection should follow standard CAT management principles, favoring LMWH or DOACs, with fondaparinux or UFH reserved for selected cases.
- Invasive interventions may be considered in carefully selected patients. Thrombolysis is generally reserved for patients with severe symptoms, low bleeding risk, and intention to preserve the catheter. Thrombectomy should only be considered in specific clinical contexts and after failure of conservative management.

### 3.3. Upper Extremity DVT

Upper extremity DVT (UEDVT) is uncommon outside the setting of CVCAT or in the absence of compression of vascular structures. It involves the brachiocephalic vein, as well as the subclavian, axillary, internal jugular, and brachial veins, and may also affect the superior vena cava [4]. These thrombotic events are associated with recurrence, bleeding complications, and mortality rates ranging from 5.1 to 9.8%, 3.1 to 6.7%, and 24 to 35%, respectively, according to prospective and retrospective studies [4]. At presentation, patients with UEDVT tend to have lower clinically evident PE than those with lower extremity DVT (9% vs. 29%, OR 0.24), although prognosis at 3 months appears to be similar. Compared with CVC-AT, UEDVT is associated with higher rates of major bleeding, recurrent VTE, and mortality [4].

Management is generally similar to that of lower extremity DVT, including indications for anticoagulation and thrombolysis [4]. In cases in which anticoagulation is contraindicated, patients should undergo close clinical follow-up until the contraindication resolves or thrombus progression occurs, at which point the risk–benefit balance of initiating anticoagulation should be reassessed [4].

When selecting anticoagulant therapy, the same principles used for other DVT should be applied. Although the ESMO guidelines state that clinical experience with DOACs in thrombosis at unusual sites remains limited [2], it should be noted that the ADAM-VTE clinical trial evaluating apixaban included patients with UEDVT, making it the only randomized controlled trial of a DOAC to clearly include this population [31]. Regarding other anticoagulation studies, the following information is available:

- The ONCENOX study evaluating enoxaparin did not specify the types of thrombosis included, referring only to patients diagnosed with VTE [32]; therefore, patients with UEDVT may have been included.
- Most RCTs included patients with proximal DVT and/or PE, thereby excluding patients with UEDVT. These studies included CLOT (dalteparin) [33], CATCH (tinzaparin) [34], HOKUSAI (edoxaban) [35], SELECT-D (rivaroxaban) [36], and CARAVAGGIO (apixaban) [37]. The CANTHANOX study evaluating enoxaparin explicitly excluded patients with UEDVT [38].
- The fondaparinux study for DVT also focused exclusively on lower extremity DVT [39].
- The API-CAT study, which evaluated extended anticoagulation with low-dose apixaban, included only patients with proximal DVT and/or PE [40].
- Other non-pivotal studies assessed include:
  ○ The ELEBAMA study evaluating bemiparin, which included only patients with proximal DVT and/or PE [41].
  ○ The TREBECA study evaluating LMWH (enoxaparin, tinzaparin, bemiparin) included patients with VTE, of whom 14.8% had UEDVT. It was not specified whether these cases were CVCAT; however, only 10% of patients had a CVC, suggesting the presence of non-CVC-associated UEDVT [42].

In summary, very few clinical trials have specifically included patients with UEDVT in their eligibility criteria, with ADAM-VTE being the only study to clearly do so, and ONCENOX potentially including these patients. In addition, an observational study evaluating LMWH included these patients. Therefore, although evidence remains limited, there is no clear rationale for managing this form of VTE differently from others, including indications for thrombolysis or thrombectomy. Notably, among the major clinical guidelines reviewed, only the NCCN guidelines specifically address the management of UEDVT [4].

### 3.4. Splanchnic Venous Thrombosis

Among the guidelines reviewed, only the NCCN address this entity. Therefore, the information presented in this section (unless otherwise specified) is derived from this guideline [4].

Splanchnic venous thrombosis (SPVT) includes thrombosis of the hepatic veins (Budd–Chiari syndrome), portal vein, mesenteric veins, and splenic vein, and is associated with reduced survival. SPVT may present as isolated or multiple thromboses, with isolated portal vein thrombosis being the most common presentation. Multiple SPVT has been associated with worse 10-year survival compared with isolated thrombosis (48% vs. 68%). In patients with extrahepatic portal vein thrombosis, the presence of synchronous mesenteric vein thrombosis has also been associated with reduced survival. Some retrospective studies have reported a 30-day mortality rate of approximately 20% in cases of mesenteric thrombosis. In addition, mesenteric thrombosis may lead to intestinal infarction in up to 30–45% of cases, with approximately 19% of these being fatal [4].

In addition to cancer, risk factors for SPVT include recent abdominal surgery, abdominal mass, pancreatitis, cirrhosis, paroxysmal nocturnal hemoglobinuria, myeloproliferative neoplasms, and JAK2V617F mutations. The use of exogenous estrogens has also been implicated [4].

Symptoms of acute SPVT commonly include abdominal pain (colicky or non-colicky), abdominal distension, peritoneal irritation, guarding, fever, anorexia, nausea, vomiting, diarrhea, gastrointestinal bleeding, hepatomegaly, and ascites, although SPVT may also be detected incidentally. Peritoneal irritation, guarding, and fever may suggest intestinal infarction. Chronic SPVT is often asymptomatic because of the development of collateral circulation, although symptoms such as abdominal pain, nausea, vomiting, anorexia, lower extremity edema, and splenomegaly have been described. Chronic mesenteric thrombosis may additionally be associated with weight loss, abdominal distension, and postprandial pain. The presence of splenomegaly and/or esophageal varices suggests portal hypertension (PHT) associated with chronic SPVT. Acute SPVT is generally defined by symptom duration ≤ 8 weeks in the absence of portal cavernoma or signs of PHT [4].

Diagnosis of acute SPVT includes complete blood count, biochemistry, and coagulation studies. Imaging modalities include ultrasound, contrast-enhanced abdominal/pelvic CT, and contrast-enhanced abdominal MRI. When hepatic and/or portal vein involvement is suspected, Doppler ultrasound is generally considered the imaging modality of choice, whereas CT is preferred in cases of mesenteric involvement because of interference from intestinal gas. If imaging studies are negative or inconclusive, alternative diagnoses should be investigated. If cases of persistent clinical suspicious, repeat imaging should be considered after discussion with radiology to optimize test selection [4].

Treatment varies according to the location and presentation of SPVT.

#### 3.4.1. Acute Hepatic Vein Thrombosis

Acute hepatic vein thrombosis is defined by symptom duration ≤ 8 weeks. Patients should undergo gastroenterology evaluation, and anticoagulation should be initiated unless contraindicated. In patients with severe symptoms or PHT, referral to specialized centers for pharmacomechanical thrombectomy with or without transjugular intrahepatic portosystemic shunt (TIPS) is recommended [4].

The choice of intervention, including thrombolysis or shunting, should be multidisciplinary and individualized according to local expertise, thrombus characteristics and bleeding risk. If anticoagulation is contraindicated, patients should be managed with TIPS or surgical shunts, with a continuous reassessment of contraindications to anticoagulation [4].

#### 3.4.2. Chronic Hepatic Vein Thrombosis

Chronic hepatic vein thrombosis is defined by symptom duration > 8 weeks. Patients should undergo gastroenterology evaluation, and TIPS (in cases of PHT) or surgical shunt should be considered in addition to anticoagulation. The risks and benefits of anticoagulation must be carefully assessed. Duration should be at least 6 months for provoked events (e.g., surgery) and indefinite in unprovoked cases, active cancer, or persistent thrombophilia [4].

#### 3.4.3. Acute Portal, Mesenteric, and/or Splenic Vein Thrombosis

As with hepatic veins, acute portal, mesenteric, and/or splenic vein thrombosis is defined by symptom duration ≤ 8 weeks, without cavernous transformation/collaterals or signs of PHT. In patients without contraindications to anticoagulation, management is similar to that recommended for acute hepatic vein thrombosis [4].

Acute mesenteric thrombosis carries a high risk of intestinal infarction, which is life-threatening and requires urgent surgery to resect necrotic bowel segments. If anticoagulation is contraindicated, patients should undergo periodic reassessment and evaluation by gastroenterology and surgical teams, with surgical management indicated if intestinal infarction develops [4].

#### 3.4.4. Chronic Portal, Mesenteric, and/or Splenic Vein Thrombosis

Chronic portal, mesenteric, and/or splenic vein thrombosis is defined by symptom duration > 8 weeks or by the presence of cavernous transformation/collaterals or signs of PHT. As in acute cases, patients should undergo gastroenterology evaluation and surgical assessment if intestinal infarction is suspected. Management includes beta-blockers, esophageal variceal banding or sclerosis, and TIPS or surgical shunting, together with anticoagulation under the same considerations as chronic hepatic vein thrombosis [4].

#### 3.4.5. Anticoagulant Therapy

Anticoagulant treatment follows the same principles as for other VTEs regarding choice of agent and contraindications. A minimum treatment duration of 6 months is recommended, although the role of long-term treatment remains controversial. An individual patient meta-analysis reported reduced risk of SPVT recurrence (HR 0.42, 95% CI 0.27–0.64), major bleeding (HR 0.47, 95% CI 0.30–0.74), and mortality (HR 0.23, 95% CI 0.17–0.31) during anticoagulation compared with untreated periods; notably, 32% of included patients had solid tumors. Conversely, a retrospective cohort study found no benefit of anticoagulation in preventing SPVT recurrence. An important limitation of this retrospective study was the use of warfarin, which is generally not recommended in oncology patients, thereby limiting extrapolation of these findings to this population [4].

In patients with chronic SPVT, the presence of PHT may further increase bleeding risk because of esophageal varices or splenomegaly-associated thrombocytopenia. Consequently, the NCCN guidelines conclude that, in the absence of RCT, decisions regarding long-term anticoagulation should be individualized [4].

The SEMI-SEOM-SETH consensus also discusses whether incidental SPVT should receive anticoagulation [5]. This consensus references an ISTH registry that was not limited to patients with solid tumors and included 177 incidental SPVT cases, of which 62 (35%) occurred in patients with non-hematologic malignancies. In this subgroup, one major bleeding event (1.2 cases/100 patient-years) and seven thrombotic recurrences (8.1 cases/100 patient-years) were reported. Additional findings included [5]:

- A low likelihood of anticoagulant use in both incidental SPVT and cancer patients.
- Marked heterogeneity in anticoagulant regimens and treatment duration, ranging from 6 months of LMWH therapy to 224 months with VKA treatment.
- Patients with platelet counts < 100,000/μL were less likely to receive anticoagulation and had the highest rates of major bleeding. Thrombotic recurrence was more frequent in men with incidental thrombosis and short anticoagulation duration.
- Regardless of cancer status, recurrence rates during anticoagulation were similar between symptomatic and incidental SPVT.
- During anticoagulation, major bleeding rates did not exceed thrombotic recurrence rates, although specific oncology data were lacking.

A RIETE study including 309 patients with incidental SPVT (59% of the total) reported that most patients received anticoagulation. Compared with symptomatic SPVT, incidental SPVT was associated with a non-significantly higher risk of symptomatic recurrence, whereas rates of major bleeding were similar. Active cancer was associated with increased thrombotic recurrence.

The SEMI-SEOM-SETH consensus acknowledges that, despite the low-quality of available evidence, anticoagulation is recommended in cancer patients without contraindications, with individualized decision-making, similarly to NCCN recommendations [4,5]. Unlike the NCCN guidelines, this consensus does not recommend routine endoscopic evaluation to exclude esophageal varices and does not provide specific recommendations according to the affected splanchnic vein [5]. Although NCCN guidelines distinguish hepatic veins from other splanchnic veins, overall recommendations are largely similar [4]. While anticoagulation should generally be considered in all patients according to individual characteristics, the SEMI-SEOM-SETH consensus suggests that anticoagulation may be particularly justified in patients with portal vein thrombosis awaiting liver transplantation or in superior mesenteric vein thrombosis involving extensive bowel segments [5]. Finally, the SEMI-SEOM-SETH consensus differs from NCCN recommendations by suggesting a minimum anticoagulation duration of 3 months [5], whereas the NCCN guidelines recommend at least 6 months or indefinite anticoagulation in selected cases [4].

### 3.5. Anticoagulant Therapy in Cancer Patients with Thrombocytopenia

Thrombocytopenia is common among patients with cancer, particularly in those receiving chemotherapy [1]. Because thrombocytopenia may contraindicate anticoagulant therapy or thromboprophylaxis, establishing management recommendations for patients requiring these interventions is essential, especially considering that thrombocytopenia increases bleeding risk without reducing thrombotic risk [1].

There is no universal consensus among clinical guidelines regarding platelet thresholds that constitute absolute or relative contraindications to anticoagulation. Thrombocytopenia should be considered alongside other relevant factors, including its underlying cause, expected duration, tumor-related vascular involvement, renal or hepatic impairment, prior bleeding history, coagulopathies, and other patient-specific characteristics. In general, for the use of therapeutic-dose anticoagulation, a platelet count < 20,000–25,000/μL is considered an absolute contraindication, whereas platelet counts < 50,000/μL are generally considered a relative contraindication (absolute in the case of DOACs). There is broad agreement that full-dose anticoagulation can be safely administered when platelet counts are >50,000/μL [1,2,3,4,5,6,8,9,10]. Greater controversy exists regarding platelet thresholds for in-hospital prophylaxis, with the ITAC guidelines being the most restrictive [3]. No guideline provides specific recommendations for outpatient prophylaxis in patients with thrombocytopenia; however, the AVERT and CASSINI trials included patients with platelet counts as low as 50,000/μL [43,44,45].

These thresholds are summarized in Table 1 according to current clinical guidelines.

#### Management Recommendations

Regarding thromboprophylaxis in hospitalized patients, most guidelines do not provide specific management recommendations in the presence of thrombocytopenia. The NCCN guidelines state that, in high-risk patients, prophylaxis may be considered with platelet counts as low as 25,000/μL [4]. In patients not considered high risk, mechanical prophylaxis should be considered. No guideline specifically addresses outpatient prophylaxis management in patients with thrombocytopenia; therefore, in this setting, we consider discontinuing of anticoagulation when platelet count falls below 50,000/μL to be the most reasonable approach.

Clinical guidelines do not define platelet thresholds contraindicating outpatient prophylaxis; therefore, exclusion criteria from relevant clinical trials were considered. In the PROTECTH, TOPIC-1 and TOPIC-2 trials, patients with platelet counts < 75,000/μL were excluded [46,47,48], whereas the CONKO-004 trial allowed dose adjustments in patients with platelet counts of 50,000–75,000/μL [49]. Similarly, the AVERT and CASSINI trials excluded patients with platelet counts < 50,000/μL [43,44,45].

For patients requiring therapeutic anticoagulation with platelet counts < 50,000/μL, several guidelines recommend balancing bleeding risk against the risk of VTE recurrence [1,4]. Bleeding risk assessment should consider the need for invasive procedures, as well as the cause, severity, and anticipated duration of thrombocytopenia. Additional factors include renal or hepatic impairment, tumor-related vascular invasion, prior bleeding history, and coagulopathies [1,4].

LMWH is generally preferred over DOACs or VKA because of greater clinical experience, shorter half-life, and easier dose adjustment (e.g., twice-daily dosing) [1]. Individualized assessment of both bleeding and recurrence risk, as well as the anticoagulant previously used, is recommended.

Several guidelines stratify patients according to the risk of VTE recurrence to guide management decisions, although some classify patients according to acute versus subacute VTE. Definitions of high- and low-risk disease differ slightly among guidelines:

- High risk:
  ○ Any VTE occurring within 1 month of the index event [1,2,5,6].
    ▪ NCCN restricts this category to PE and proximal DVT [4].
  ○ PE with high thrombotic burden [1,2]; ESMO defines this as segmental or more proximal.
  ○ Proximal DVT [1,2].
  ○ History of recurrent thrombosis [2,4].
- Low risk:
  ○ CVCAT [1,4].
  ○ Distal DVT > 1 month after the index event [1,2].
    ▪ NCCN considers any distal DVT to be low risk [4].
  ○ Any VTE > 1 month after the event [4,5,6].
  ○ Isolated subsegmental PE [2].
  ○ UEDVT [4].

Given the discrepancies among guidelines, and the potential for contradictory recommendations in certain scenarios, we propose the following simplified approach:

- High risk:
  ○ History of recurrent thrombosis.
  ○ VTE occurring < 1 month from the index event (acute phase). Depending on context and bleeding risk, distal DVT occurring within the first month, CVCAT, and UEDVT may be considered lower risk.
- Low risk:
  ○ VTE occurring > 1 month from the index event (subacute phase). Depending on context and bleeding risk, relatively recent (<3 months) proximal DVT or high-burden PE may still be considered high risk.
  ○ CVCAT.
  ○ UEDVT.

Based on recurrence risk stratification, several clinical guidelines (SEOM, ESMO, NCCN, SEMI and Spanish expert consensus) provide recommendations according to platelet counts [1,2,4,6,10]. These recommendations include consideration of platelet transfusion, although the SEMI-SEOM-SETH consensus is more restrictive regarding this strategy because it may be difficult to maintain, may fail to achieve target platelet counts, and is associated with potential adverse effects [5].

These recommendations are summarized in Table 2.

Regarding IVCF placement, guidelines provide heterogeneous recommendations. In high-risk patients with platelet counts between 25,000 and 50,000/μL, the NCCN guidelines consider IVCF placement a potential alternative [4], whereas other guidelines reserve it for cases of contraindication to anticoagulation or inability to maintain platelet counts despite transfusions support [2,6,10]. In high-risk patients with platelet counts < 25,000/μL, IVCF placement may also be considered [2,4,6]. The IVCF should be maintained until resolution of thrombocytopenia and/or contraindication. The SEMI-SEOM-SETH consensus additionally considers IVCF placement when platelets < 20,000/μL or in patients with poor cardiopulmonary reserve, particularly when thrombocytopenia is expected to last >5–7 days [5].

Overall, we favor the approach by SEOM, ESMO, NCCN, and SEMI. However, the SEMI-SEOM-SETH strategy may be considered when platelet transfusion is not feasible and platelet counts range between 25,000 and 50,000/μL or in cases of very high thrombotic risk with platelets counts <25,000/μL when IVCF placement is not possible.

### 3.6. Central Nervous System (CNS) Involvement: Primary Tumors and Metastases

CNS involvement by tumor (primary or metastatic) is not considered a contraindication to anticoagulation in several guidelines [1,8,9], although there are concerns regarding intracranial hemorrhage (ICH). The NCCN guidelines consider CNS involvement a relative contraindication but do not provide specific management recommendations [4].

Although prospective data are lacking, observational studies and meta-analyses have shown that anticoagulated patients with CNS involvement have higher risk of ICH [50,51,52] and fatal ICH [50] than anticoagulated patients without CNS disease. The magnitude of this risk appears to vary according to tumor type, with brain metastases showing a higher risk of ICH than gliomas [51,53]. In patients with brain metastases, anticoagulation does not appear to significantly increase ICH risk according to two meta-analyses and one cohort study [51,53,54,55], whereas an increased risk has been observed in patients with glioma [51,53,54,56].

Melanoma and renal cell carcinoma brain metastases show higher baseline rates of ICH. However, subgroup analyses suggest that anticoagulation does not significantly increase ICH incidence compared with non-anticoagulated patients [54,55].

Regarding anticoagulant selection, patients with gliomas treated with DOACs appear to have lower rates of overall and major ICH than those treated with LMWH [51,53,57,58], with no significant differences observed among patients with brain metastases [53]. Although evidence in patients with brain metastases remain controversial—with some studies suggesting a lower risk of ICH with DOACs [51] and others showing no significant difference [57,58]—most clinical guidelines currently recommend full-dose anticoagulation regardless of whether the CNS tumor is primary or metastatic [1,3,5,8,9]. Given the favorable safety profile observed in several meta-analysis [51,57,58], we believe that DOACs should be prioritized over LMWH in patients with glioma in the absence of contraindications.

Routine dose reduction is generally not recommended, unless additional factors are present (e.g., thrombocytopenia, renal impairment). However, the SEMI-SEOM-SETH consensus suggests reducing the LMWH dose by 25–50% in patients with melanoma or renal cell carcinoma brain metastases, particularly in non-severe VTE. The same recommendation is made for glioma patients until local disease control is achieved, after which full-dose LMWH may be resumed [5]. Similar recommendations are included in the Spanish expert consensus, extended to brain metastases from thyroid cancer, choriocarcinoma, and hepatocellular carcinoma metastases although these recommendations are weak and based on moderate-quality evidence [10].

The ASCO guidelines advise against IVCF placement solely to avoid anticoagulation in patients with glioma [8,9], as filters are associated with complications and have not been shown to reduce VTE incidence [54].

The ITAC guidelines discourage outpatient thromboprophylaxis in patients with non-operated brain tumors and are the only guidelines to provide specific recommendations in this setting [3].

In summary, substantial variability can be observed across clinical guideline recommendations, and in some cases, recommendations are lacking altogether. Most guidelines emphasize the need for individualized decision-making, although they frequently provide limited practical guidance to support such decisions.

**Figure 1:**
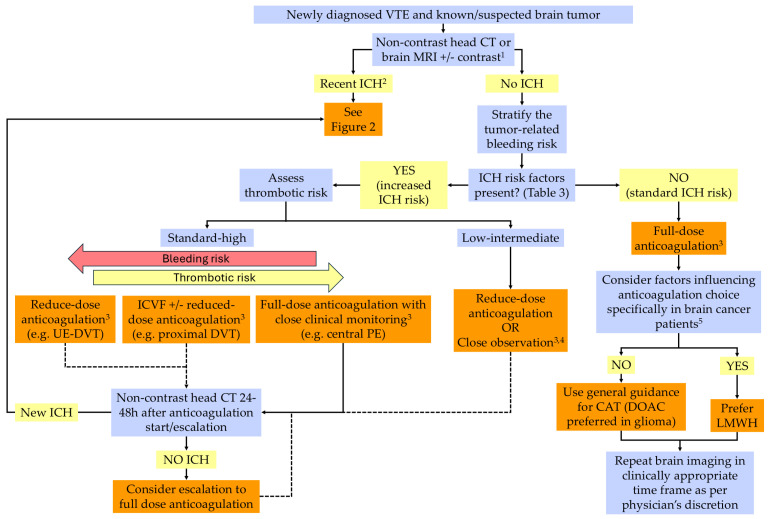
Management of acute VTE in patients with brain cancer, without recent ICH (adapted by Leader et al. [59]). ^1^ If no imaging within 2 weeks. ^2^ Recent ICH defined as imaging evidence of ICH in past 30 days. Hemosiderin residues (usually intratumoral) are not considered to be recent ICH. ^3^ Discuss risk–benefit ratio of anticoagulation and alternatives with patient and/or health care proxy and document before treatment initiation. ^4^ Close observation without anticoagulation includes lower extremity Doppler ultrasound after 1 week (consider repeat in 2 weeks) to exclude new DVT or proximal DVT progression. ^5^ Phenytoin, carbamazepine or phenobarbital treatment; dynamic thrombocytopenia; oral intake issues; impending surgery. VTE: venous thromboembolism event; CT: computed tomography; MRI: magnetic resonance imaging; ICH: intracranial hemorrhage; UE: upper extremity; DVT: deep venous thrombosis; ICVF: inferior vena cava filter; PE: pulmonary embolism; CAT: cancer associated thrombosis; DOAC: direct oral anticoagulant; LMWH: low-molecular-weight heparin.

In this context, we consider the work by Leader et al. to be particularly valuable, as they proposed a management algorithm for patients with cerebral involvement, including both primary brain tumors and brain metastases. This algorithm is summarized in Figure 1, which was adapted from the work of Leader et al. [59].

As shown in this algorithm, the authors recommend performing an initial brain imaging assessment to determine the presence or absence of ICH. In the absence of ICH, patients should subsequently undergo stratification according to their risk of developing ICH. To support this assessment, the authors propose a series of risk factors, summarized in Table 3, which was adapted from the model presented by Leader et al. [59]. The authors also acknowledge that this risk assessment remains a complex and challenging task.

Once the risk of ICH has been established, patients without additional risk factors should generally be managed according to the standard recommendations for the treatment of VTE, with two important considerations. First, the algorithm highlights the need to account for factors that may influence anticoagulant selection, particularly concomitant medications commonly used in this population, such as phenytoin, which may interact with DOACs. In such cases, LMWH may be preferred. Second, in the absence of other factors influencing anticoagulant choice, DOACs are preferred in patients with gliomas because of their favorable safety profile in this setting [59].

The second step, following the assessment of ICH risk, concerns patients considered to be at increased risk of developing ICH. In this setting, thrombotic risk should be carefully evaluated before treatment decisions are made. In patients with low-to-intermediate thrombotic risk, the algorithm proposes the use of reduced-dose anticoagulation together with close clinical monitoring. According to the authors, low-to-intermediate-risk events include isolated distal DVT, isolated subsegmental PE, and other lower-risk thrombotic events, such as CVC-AT.

In patients with standard or high thrombotic risk, a careful balance between thrombotic and bleeding risks is required, as well as consideration of the type and location of thrombosis. In cases of central PE, full-dose anticoagulation may still be considered, whereas in patients with proximal DVT, placement of an IVCF together with reduced-dose anticoagulation, or even without anticoagulation, may be an option. In situations where an IVCF is unlikely to provide clinical benefit, such as UE-DVT, reduced-dose anticoagulation may be considered instead [59].

Importantly, Leader et al. do not specify the degree of anticoagulant dose reduction [59]. From our perspective, intermediate-dose LMWH or prophylactic-dose anticoagulation could be reasonable options, although it should be acknowledged that evidence supporting this specific approach remains limited.

The authors also propose that, in all patients with an increased risk of ICH who initiate anticoagulation, a non-contrast head computed tomography (CT) scan should be performed 24–48 h after treatment initiation to assess for the occurrence of ICH. In the absence of ICH, escalation to full-dose anticoagulation may subsequently be considered on a case-by-case basis, with repeat neuroimaging performed following dose escalation [59].

#### Hemorrhagic Brain Metastases

Active bleeding constitutes a major contraindication to VTE prophylaxis [1,2,4,5]. Regarding therapeutic anticoagulation, major active bleeding (including ICH) is considered an absolute contraindication by several guidelines [4,8,9], or a relative contraindication if the bleeding event occurred within the previous 4 weeks [8,9]. Although no specific recommendations are available regarding VTE management in patients with hemorrhagic brain metastases in clinical guidelines, the presence of active ICH would generally be considered an absolute contraindication to anticoagulation in this population.

First, it is important to define what should be considered an ICH and how it should be assessed. Leader et al. note that both CT and magnetic resonance imaging (MRI) provide relevant information regarding the timing and volume of bleeding [59]. Non-contrast CT is typically the initial imaging modality because of its wide availability and accessibility. Hyperdense blood products reach peak density on CT within the first few hours after ICH and gradually become more isodense over the following days to weeks as blood resorption occurs [59].

Differentiating ICH from an underlying mass lesion on CT may be challenging, as both primary and metastatic brain tumors can appear hypo-, iso-, or hyperdense regardless of the presence of hemorrhage. In addition, intratumoral calcifications may also appear hyperdense and mimic small punctate hemorrhagic foci. In this context, CT brain imaging in monitoring intratumoral hemorrhage is the most useful over serial scans, in which the relative density of the underlying mass has been established [59].

MRI, on the other hand, provides greater accuracy in determining the temporal stage of ICH because of the different paramagnetic properties associated with the evolving states of hemoglobin degradation (oxyhemoglobin, deoxyhemoglobin, methemoglobin, ferritin, and hemosiderin). This is especially relevant when evaluating the safety of anticoagulation according to the chronicity of the hemorrhage. In cases of mild ICH, MRI can distinguish between hyperacute (<1 day), acute (1–3 days), early subacute (3–7 days), late subacute (7–28 days), and chronic (>28 days) hemorrhage [59].

It should also be recognized that cystic cavities within tumors may contain layered blood products, particularly in highly hemorrhagic neoplasms such as melanoma and renal cell carcinoma. Moreover, hemorrhage within brain tumors is frequently heterogeneous in age, as these lesions may bleed repeatedly over time, often in association with additional contributing factors such as coagulopathies or anticoagulant therapy [59].

Finally, small-volume, asymptomatic hemosiderin deposits are frequently observed in brain tumors. Although these findings do not constitute an absolute contraindication to anticoagulation on their own, they should be carefully monitored. In this regard, review of imaging studies with a neuroradiologist may be particularly useful to support longitudinal assessment and guide anticoagulation-related decision-making [59].

Management should therefore be individualized, with careful assessment of the risks and benefits of anticoagulation. Currently, no clinical guidelines provide specific recommendations regarding anticoagulant selection or dosing in this scenario, although a cautious multidisciplinary approach is generally advised. Leader et al. also proposed a decision-making algorithm for patients who experienced an ICH within the previous 30 days, as prior ICH is considered a risk factor for recurrent bleeding. In this algorithm, the authors first recommend an assessment of thrombotic risk. Isolated distal DVT and isolated subsegmental PE are considered lower-risk thrombotic events, and anticoagulation should generally remain withheld in these situations. In contrast, in patients with PE or proximal DVT, placement of an IVCF may be considered, whereas this strategy is not routinely recommended for thrombosis in other locations. However, regarding IVCF placement, the authors also acknowledge that the evidence supporting this approach remains limited [59].

After thrombotic risk assessment, the authors propose evaluating the extent of the previous hemorrhage, considering a conservative threshold of 10 mL of ICH volume as a clinically reasonable cutoff when deciding whether anticoagulation may be resumed. Accordingly, anticoagulation is discouraged in patients with ICH volumes > 10 mL. In contrast, prophylactic-dose anticoagulation may be considered in patients with ICH volumes < 10 mL who are deemed to be at high risk of thrombotic complications [59].

As in patients without prior ICH, repeat non-contrast head CT is recommended 24–48 h after anticoagulation initiation to assess for hemorrhagic progression, with the possibility of dose escalation in the absence of imaging or clinical complications [59].

**Figure 2:**
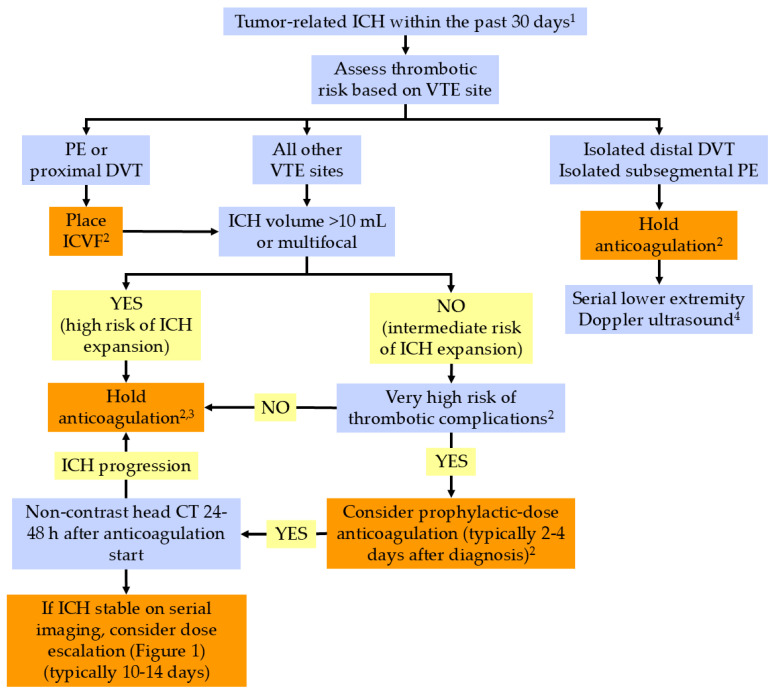
Management of acute VTE in patients with brain cancer, with recent ICH (adapted by Leader et al. [59]). ^1^ Excluding hemosiderin residues (usually intratumoral) as the sole finding. ^2^ Discuss risk–benefit ratio of anticoagulation and alternatives with patient and/or health care proxy and document before treatment initiation. ^3^ Periodically reassess risk–benefit ratio of initiating anticoagulation. ^4^ Lower extremity Doppler ultrasound after 1 week (consider repeat in 2 weeks) to exclude new DVT or proximal DVT progression. ICH: intracranial hemorrhage; VTE: venous thromboembolism event; PE: pulmonary embolism; DVT: deep venous thrombosis; ICVF: inferior vena cava filter; CT: computed tomography.

These recommendations are summarized in the algorithm shown in Figure 2, which was adapted from the model proposed by Leader et al. [59].

### 3.7. Patients Receiving Antiangiogenic Therapy

Evidence on this topic is limited, and most clinical guidelines do not provide specific recommendations. The SEMI-SEOM-SETH consensus is the only guideline that specifically addresses this issue. It does not recommend routine anticoagulant dose reduction in patients receiving antiangiogenic agents, although caution is advised in patients with CNS involvement [5].

Following a VTE requiring anticoagulation, antiangiogenic therapy should generally be withheld for approximately 2 weeks before resumption. In cases of life-threatening VTE, permanent discontinuation of antiangiogenic therapy is recommended [5].

The Spanish expert consensus provides additional recommendations for VTE occurring during antiangiogenic therapy stratified according to Common Terminology Criteria for Adverse Events (CTCAE) v4.03 severity. These recommendations largely overlap with those established by the SEMI-SEOM-SETH consensus, although several additional considerations are included [10]:

- Grade 3 VTE (thrombosis [e.g., uncomplicated PE, non-embolic cardiac mural thrombus], medical intervention indicated): Interrupt antiangiogenic therapy and initiate anticoagulation. Therapy may be resumed after approximately 2 weeks. Do not resume in cases of prior antiangiogenic-related bleeding or tumor invasion of major vessels.
- Grade 4 VTE (life-threatening events [e.g., PE, cerebrovascular event, arterial insufficiency]; hemodynamic or neurologic instability; urgent intervention indicated): Permanent discontinuation of antiangiogenic therapy.
- Arterial thrombosis: Permanent discontinuation of antiangiogenic therapy.
- Recurrent or progressive thrombosis despite adequate anticoagulation: Permanent discontinuation of antiangiogenic therapy.

Additional recommendations include optimization of cardiovascular risk factors before initiation of antiangiogenic therapy, cautious use in patients with arterial thrombotic events within the previous 6–12 months (although this is not considered an absolute contraindication), consideration of low-dose aspirin prophylaxis in high-risk patients and in those with history of arterial thrombosis, and cautious reintroduction in selected cases when the potential clinical benefit outweighs the risks and the patient has been adequately informed.

### 3.8. Renal Impairment

Renal function should be assessed before initiation of anticoagulant therapy, as patients with renal impairment are at increased risk of major bleeding and recurrent VTE during anticoagulation [1,2,5]. Patients with a creatinine clearance (CrCl) > 30 mL/min should receive full-dose anticoagulation and may be treated with LMWH or DOACs [1,2,4]. Fondaparinux may be used with caution in patients with CrCl 30–50 mL/min [4].

Importantly, patients with CrCl < 30 mL/min were excluded from pivotal clinical trials. In general, LMWH or UFH is recommended in patients with CrCl 15–30 mL/min, whereas fondaparinux is contraindicated [60]. Although some clinical guidelines do not address DOAC use in this setting [1] and others discourage it [2,4], the corresponding SmPCs permit their use and provide dosing recommendations. Indeed, ASCO guidelines recommend referring to the SmPCs for guidance [8,9].

Recommendations for primary prophylaxis (both hospitalized and ambulatory patients) and the treatment of VTE are summarized below. These recommendations are derived from clinical practice guidelines and/or SmPC indications. In all cases, anticoagulation in patients with renal impairment requires close monitoring of renal function and bleeding risk. Unless otherwise specified, the following recommendations apply to patients with CrCl < 30 mL/min.

#### 3.8.1. Primary Prophylaxis in Hospitalized Patients

- Fondaparinux: Contraindicated [4,60].
- History of HIT: Mechanical prophylaxis is recommended. Although not specifically addressed in all guidelines, several recommend mechanical prophylaxis when pharmacologic prophylaxis is contraindicated; this recommendation would apply in this scenario, as both LMWH and UFH are contraindicated [1,2,3,4,5].
- CrCl 15–30 mL/min: LMWH prophylaxis may be considered using dose-adjusted regimens based on available guidelines and SmPC recommendations. Enoxaparin may be administered at 20 mg subcutaneously (SC) once daily according to the SmPC [61], whereas NCCN guidelines recommend 30 mg once daily [4]. Tinzaparin may be used at a dose of 4500 IU SC once daily in patients with CrCl ≥ 20 mL/min, while its use is contraindicated in those with CrCl < 20 mL/min [10,62,63]. Bemiparin may be administered at 2500 IU CS once daily, with consideration of anti-Xa monitoring, as recommended in the SmPC [64,65]. In contrast, the use of dalteparin is generally discouraged in this setting [4,66]. UFH may also be considered and is the preferred option in the NCCN guidelines, although its use is more complex because of the need for closer monitoring [3,4]. Some guidelines additionally consider mechanical thromboprophylaxis in selected patients [3].
- CrCl < 15 mL/min: LMWH is contraindicated [61,62,63,64,65,66]. UFH 5000 IU SC every 8–12 h is recommended [4].

**Figure 3:**
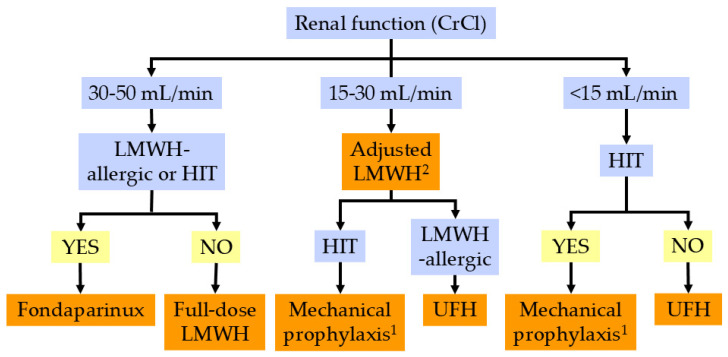
Algorithm for choosing an anticoagulant for antithrombotic prophylaxis in hospitalized patients and renal impairment. ^1^ Check for possible contraindications. ^2^ Enoxaparin and bemiparin require dose adjustment. Tinzaparin does not require dose adjustments with CrCl ≥ 20 mL/min and is contraindicated if <20 mL/min. Use of dalteparin is discouraged. CrCl: creatinine clearance; LMWH: low-molecular-weight heparin; HIT: heparin-induced thrombocytopenia; UFH: unfractionated heparin.

An algorithm for choosing the anticoagulant in this scenario is proposed in Figure 3.

#### 3.8.2. Primary Prophylaxis in Ambulatory Patients

The NCCN guidelines are the only guidelines that specifically address this scenario [4]. They recommend applying the same criteria used for hospitalized patients and advise against the use of dalteparin, fondaparinux, and rivaroxaban in patients with CrCl < 30 mL/min. Although NCCN states that the recommendations are the same as for hospitalized patients, it discourages enoxaparin use for outpatient prophylaxis when CrCl < 30 mL/min [4]. We believe that enoxaparin may still be considered with appropriate dose adjustment:

- CrCl 15–30 mL/min:
  ○ DOACs: In this setting, selected DOACs may be considered with caution [4]. Apixaban can be used without dose adjustment, particularly in patients with a history of HIT [67]. Similarly, the rivaroxaban SmPC states that no dose adjustments are required at this level of renal function [68].
  ○ LMWH: In this setting, the same LMWH recommendations described for prophylaxis of hospitalized patients should be followed.
- CrCl < 15 mL/min: No specific recommendations available for outpatient prophylaxis in patients with this degree of renal impairment. However, outpatient prophylaxis should generally be avoided due to the difficulty of close renal monitoring and because the SmPCs contraindicate use at this level of renal function [61,62,63,64,65,66,67,68].

**Figure 4:**
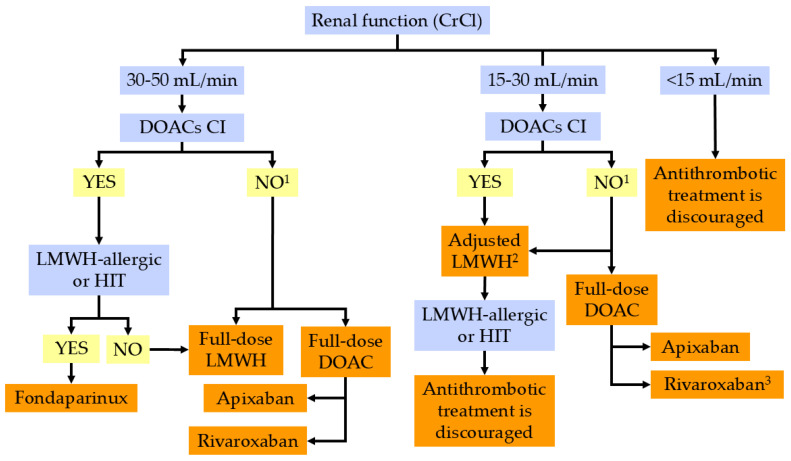
Algorithm for choosing an anticoagulant for antithrombotic prophylaxis in ambulatory patients and renal impairment. ^1^ In the absence of CI to DOACs, both these and LMWH can be prescribed, considering the patient’s characteristics and preferences. ^2^ Enoxaparin and bemiparin require dose adjustment. Tinzaparin does not require dose adjustments with CrCl ≥ 20 mL/min and is CI if <20 mL/min. Use of dalteparin is discouraged. ^3^ NCCN guidelines discourage its use with this level of renal impairment, but the SmPC indicates that can be used. CrCl: creatinine clearance; DOAC: direct oral anticoagulant; CI: contraindications/contraindicated; LMWH: low-molecular-weight heparin; HIT: heparin-induced thrombocytopenia; SmPC: summary of product characteristics.

An algorithm for anticoagulant selection in this setting is proposed in Figure 4.

#### 3.8.3. Treatment of VTE

- CrCl 30–50 mL/min:
  ○ Apixaban or LMWH may be used without dose adjustment [61,62,63,64,65,66,67].
  ○ Rivaroxaban [68] and edoxaban may be used with caution; edoxaban requires dose reduction to 30 mg once daily [69].
- CrCl 15–30 mL/min:
  ○ DOACs: according to the SmPC, these agents may be used with caution and may be particularly useful in patients with prior HIT:
    ▪ Apixaban: In general, no dose adjustment is required [67].
    ▪ Rivaroxaban: After the initial 3-week phase (15 mg twice daily), maintenance dosing of 15 mg (instead of 20 mg) once daily may be considered if bleeding risk outweighs recurrence risk [68].
    ▪ Edoxaban: Dose reduction to 30 mg once daily is recommended in patients with CrCl 15–50 mL/min [69]. However, given the availability of alternative agents, other options may be preferred (NCCN guidelines discourage its use with CrCl < 30 mL/min [4]).
  ○ LMWH: Dose adjustment guided by anti-Xa monitoring is recommended [1,2,3,4,5,8,9,10]. Anti-Xa levels should be measured 4–6 h after dosing, targeting approximately 1 IU/mL (therapeutic range 0.5–1.5 IU/mL) [1,2,5,66]. Initial dose reduction is advised, followed by subsequent adjustment according to anti-Xa levels:
    ▪ Enoxaparin: 1 mg/kg SC once daily [1,5,61].
    ▪ Tinzaparin: No dose adjustment required if CrCl ≥ 20 mL/min [1]; contraindicated if CrCl < 20 mL/min [10,62,63].
    ▪ Bemiparin: Reduce to 75% of the standard dose [1,5,64,65].
    ▪ Dalteparin: May be used without initial dose reduction (no specific starting dose is provided), with anti-Xa-guided adjustment recommended [66].
  ○ UFH: May be considered, particularly if anti-Xa monitoring is not feasible [1,2,3,8,9].
- CrCl < 15 mL/min: DOACs, LMWH, and fondaparinux are contraindicated [60,61,62,63,64,65,66,67,68,69]. UFH should be used, or IVCF placement may be considered [2,4,8,9].

**Figure 5:**
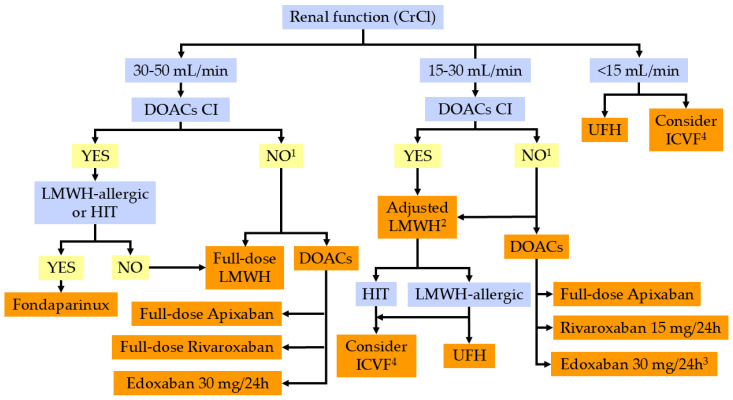
Algorithm for selection of an anticoagulant in cases of VTE and renal impairment. ^1^ In the absence of CI to DOACs, both these and LMWH can be prescribed, considering the patient’s characteristics and preferences. ^2^ Dose adjustment guided by anti-Xa monitoring is recommended (4–6 h after dosing). Initial dose reduction is advised in cases of enoxaparin and bemiparin, but not for dalteparin. Tinzaparin does not require dose adjustments with CrCl ≥ 20 mL/min and is CI if <20 mL/min. ^3^ Given the availability of alternative agents, other options may be preferred (NCCN guidelines discourage its use with CrCl < 30 mL/min [4]). ^4^ ICVF should be considered in cases where UFH is not an option. VTE: venous thromboembolism; CrCl: creatinine clearance; DOAC: direct oral anticoagulant; CI: contraindications/contraindicated; UFH: unfractionated heparin; LMWH: low-molecular-weight heparin; HIT: heparin-induced thrombocytopenia; ICVF: inferior cava vena filter.

An algorithm for anticoagulant selection in this setting is proposed in Figure 5.

### 3.9. Hepatic Impairment

Overall, evidence remains limited because these patients were excluded from most randomized controlled trials. In such cases, treatment decisions should be individualized according to the severity of hepatic impairment and the associated thrombotic and bleeding risks. Most clinical guidelines do not provide specific recommendations [3,5,6,8,9], or simply advise cautious use of anticoagulants [1,2]. According to the SmPCs of the different drugs, the following recommendations may be considered:

#### 3.9.1. LMWH

For most LMWH, available information is limited. The ESMO and SEMI clinical guidelines favor LMWH in hepatic impairment over other anticoagulants, although they provide no additional recommendation [2,6]. Drug-specific recommendations according to SmPC are as follows:

- Enoxaparin: Should be used with caution because of potential bleeding risk. The use of enoxaparin in cirrhotic patients (Child–Pugh class B-C) appears to be safe and effective for preventing portal vein thrombosis. However, caution is advised because no formal dose-finding studies have been conducted in cirrhotic patients (with any Child–Pugh class). Therefore, adjustment based on anti-Xa level monitoring is not recommended [61].
- Dalteparin: The SmPC only states that dalteparin should be used with caution in patients with severe hepatic impairment, as a factor for a greater risk of bleeding [66].
- Tinzaparin: Hepatic impairment is not mentioned as a specific concern in the SmPC [62,63].
- Bemiparin: Available data are insufficient to recommend a dose adjustment in this group of patients. “Severe hepatic function disorder” is listed as a contraindication, although the SmPC also advises caution because of an increased risk of bleeding [64,65].

#### 3.9.2. DOACs

The information provided in the SmPC of apixaban [67] and edoxaban [69] is similar, listing the following recommendations:

- Before initiation of DOACs, liver function testing should be performed.
- DOACs are contraindicated in patients with hepatic disease associated with coagulopathy and clinically relevant bleeding risk
- They are not recommended in patients with severe hepatic impairment.
- They should be used with caution in patients with mild or moderate impairment (Child–Pugh class A or B). No dose adjustments are required in patients with mild or moderate hepatic impairment.
- It should be noted that patients with ALT/AST > 2× ULN (upper limit of normality) and/or total bilirubin > 1.5× ULN were excluded from clinical trials. Therefore, DOACs should be used with caution in this population.

Regarding rivaroxaban, the information provided in the SmPC differs slightly [68], since it considers that patients with hepatic disease associated with coagulopathy and clinically relevant bleeding risk includes cirrhotic patients with Child–Pugh class B or C. In addition, adult cirrhotic patients with mild hepatic impairment (Child–Pugh class A) showed only minor pharmacokinetic changes compared with healthy controls. No specific dose adjustment recommendations are provided.

#### 3.9.3. Fondaparinux

Regarding prophylaxis, no dose adjustment is required in patients with mild-to-moderate hepatic impairment. In patients with severe hepatic impairment, it should be used with caution, as it has not been studied in this population [60].

For treatment indications, safety and efficacy have not been established in patients with severe hepatic impairment. Therefore, its use is not recommended in these patients [60].

#### 3.9.4. UFH

Therapeutic anticoagulation levels should be monitored, as in other patient populations. Although there are no specific dosage adjustment recommendations for UFH based on hepatic function, evidence suggests that its pharmacokinetics may be altered. The FDA label indicates that its clearance may be decreased, which would imply higher plasma concentrations at the same dosage [70]. Nevertheless, some studies demonstrate that patients with severe cirrhosis (Child–Pugh class C) require higher doses to achieve therapeutic anti-Xa levels. Despite requiring these higher doses, they also appear to experience increased plasma concentrations, although the difference did not reach statistical significance [71].

Furthermore, a discrepancy has been observed between aPTT and anti-Xa levels in patients with cirrhosis; specifically, supratherapeutic aPTT values may be observed despite anti-Xa levels remaining within the therapeutic range. This suggests that aPTT should not be used for monitoring this population [71,72].

The ISTH guidelines suggest that for thromboprophylaxis in hospitalized patients with cirrhosis, LMWH or fondaparinux should be preferred over UFH [73].

### 3.10. Extreme Body Weights

#### 3.10.1. Obesity

The treatment of VTE in obese cancer patients has not been extensively studied [1]. Therefore, several recommendations extrapolated from the general population have been proposed. For the purposes of these recommendations, obesity is defined as body weight > 120 kg or a body mass index (BMI) > 40 kg/m^2^.

- Prophylaxis with LMWH: Available data suggests that dose escalation is safe and effective:
  ○ Enoxaparin: 40 mg twice daily or 0.5 mg/kg once daily (an increase to 60 mg twice daily may be considered in patients with BMI > 50 kg/m^2^) [1,4,74].
  ○ Dalteparin: 7.500 IU once daily, 5.000 IU twice daily [1,4] or 40–75 UI/kg once daily [4].
- Treatment with LMWH: Dosing should be weight-adjusted without dose capping [1,2,8,9]. This recommendation is endorsed for enoxaparin [75], dalteparin [76] and tinzaparin [77].
  ○ The SEMI-SEOM-SETH consensus recommends monitoring anti-Xa levels in this population [5].
- DOACs: No dose adjustment recommendations are available according to the SmPC [67,68,69]. Currently, no evidence suggests reduced efficacy or safety in this population [78]. Clinical guidelines recommend using them with caution [1,2,8,9]. ASCO guidelines prefer LMWH over DOACs in this scenario [8,9].
- Fondaparinux:
  ○ Prophylaxis: Although no recommendations are provided in the SmPC [60], NCCN guidelines suggest considering 5 mg once daily [4].
  ○ Treatment: In patients weighing > 100 kg the recommended dose is 10 mg once daily [4,60].
- UFH: In prophylaxis, NCCN guideline recommends 7.500 IU/8 h SC [4].

#### 3.10.2. Low Body Weight

Data on patients with low body weight (25–50 kg) are limited. However, standard dosing may result in overdosing and reduced safety compared with the general population [1]. Some studies have demonstrated that lower doses of certain LMWHs are equally effective and safer in this population. Only SEOM and NCCN guidelines offer recommendations for patients with low body weight:

- Dalteparin: For prophylaxis, they recommend 2.500 IU/24 h or 100 IU/kg/24 h [1,4].
- Enoxaparin: For prophylaxis, they recommend 20 mg/24 h for weights 25–40 kg and 30 mg/24 h for weights 41–50 kg [1,4].
- DOACs: In general, clinical guidelines do not provide specific recommendations. However, NCCN guidelines discourage apixaban use in patients weighing < 40 kg [4]. The SmPC offers the following instructions:
  ○ Apixaban: Only consider lowering the dose to 2.5 mg/12 h in patients receiving apixaban for prevention of stroke and systemic embolism non-valvular atrial fibrillation and with at least two of the following criteria present: age ≥ 80 years, body weight ≤ 60 kg or serum creatinine ≥ 1.5 mg/dL. No specific recommendations are available for treatment or prevention of recurrent DVT and PE, although the SmPC warns of a potentially increased bleeding risk [67].
  ○ Rivaroxaban: The SmPC does not establish dose adjustment in adult patients, but it does for pediatric patients: it should not be used in patients < 30 kg and a dose reduction of 15 mg once daily is recommended in patients 30–50 kg [68].
  ○ Edoxaban: The SmPC recommends dose reduction to 30 mg once daily in patients weighing ≤ 60 kg [69].
- Fondaparinux:
  ○ Prophylaxis: No recommendations are provided in the SmPC [60], and NCCN guidelines consider that it is contraindicated in patients weighing < 50 kg [4].
  ○ Treatment: In patients weighing < 50 kg, the recommended dose is 5 mg once daily [4,60].
- UFH: For prophylaxis, NCCN guidelines recommend considering 2.500 IU every 8–12 h SC in patients weighing < 40 kg [4].

## 4. Conclusions

The management of CAT in special situations remains a major clinical challenge because of the lack of high-quality scientific evidence. This review addressed several complex clinical scenarios, thrombotic recurrence during anticoagulant therapy, CVC-AT, atypical localizations (upper extremity and splanchnic circulation), anticoagulant therapy in patients with thrombocytopenia, CNS involvement, concomitant antiangiogenic therapy, renal and hepatic impairment and extreme body weights. Nevertheless, additional clinical scenarios may also be considered “special situations”.

This review highlights substantial heterogeneity among the recommendations of major international guidelines, where therapeutic decisions are frequently based on expert consensus rather than robust RCT. The variability identified in key areas, including drug selection, dose adjustment in thrombocytopenia, and optimal duration of treatment, underscores the complexity of balancing the risk of recurrence against the high likelihood of bleeding complications in the oncological population.

### Future Directions

In conclusion, a more standardized and personalized therapeutic approach is needed, taking into account both tumor-specific characteristics and individual patient factors. The findings of this review emphasize the urgent need for prospective studies and controlled clinical trials specifically focused on these special situations in order to reduce diagnostic and therapeutic uncertainty. Generating high-quality evidence will be essential to optimize both safety and survival outcomes in these high-risk patients, ensuring clinical care based on scientific rigor and therapeutic efficiency.

## Figures and Tables

**Table 1:** Platelet count thresholds as contraindications to anticoagulation.

Indication	Relative CI	Absolute CI
In-hospital prophylaxis	20,000–50,000/μL ^1^ <80,000/μL ^2^	<20,000/μL ^1^ <50,000/μL ^3^
Outpatient prophylaxis ^4^	50,000–75,000/μL	<50,000/μL
Anticoagulant treatment	20,000–50,000/μL ^1,5,6^ 25,000–50,000/μL ^3,7,8^ <50,000/μL ^2^	<20,000/μL ^1,5,6^ <25,000/μL ^3,7,8^ <50,000/μL ^9^
Thrombolysis ^10^	<100,000/μL ^3^	

^1^ Recommendation made by the SEMI-SEOM-SETH consensus [5]. ^2^ Recommendation made by ITAC [3]. ^3^ Recommendation made by NCCN; however, regarding prophylaxis, they specify that in high-risk patients, it may be appropriate with platelet counts < 25,000/μL [4]. ^4^ Clinical guidelines do not specify thresholds contraindicating outpatient prophylaxis; therefore, exclusion criteria from relevant clinical trials were considered. In the PROTECTH, TOPIC-1 and TOPIC-2 studies, patients with platelet counts < 75,000/μL [46,47,48] were excluded, whereas the CONKO-004 trial allowed dose adjustments in patients with platelet counts of 50,000–75,000/μL [49]. Additionally, the AVERT and CASSINI trials excluded patients with platelet counts < 50,000/μL [43,44,45]. ^5^ Recommendations made by ASCO [8,9]. ^6^ Recommendations made by SEMI [6]. ^7^ Recommendations made by SEOM [1]. ^8^ Recommendations made by ESMO [2]. ^9^ CI applicable to DOACs, as their use is generally not recommended at platelet counts < 50,000/μL. ^10^ Systemic or catheter-directed. CI: contraindication; DOAC: direct oral anticoagulant.

**Table 2:** Recommendations for managing anticoagulant therapy in patients with thrombocytopenia.

Indication	Most Guidelines ^1^	SEMI-SEOM-SETH Consensus ^2^
25,000 ^3^–50,000/μL in high-risk VTE	100% LMWH and platelet transfusion to maintain platelet counts > 50,000/μL ^4^	50% LMWH
25,000 ^3^–50,000/μL in low-risk VTE	50% LMWH or prophylactic dose	50% LMWH
<25,000/μL ^3^ in high-risk VTE	No anticoagulation	50% LMWH and platelet transfusion to maintain platelet counts > 20,000/μL
<25,000/μL ^3^ in low-risk VTE	No anticoagulation	No anticoagulation

^1^ Recommendation made by SEOM, ESMO, NCCN and SEMI guidelines and Spanish consensus [1,2,4,6,10]. ^2^ Recommendation made by SEMI-SEOM-SETH consensus [5]. ^3^ SEMI and the SEMI-SEOM-SETH consensus use 20,000/μL as the lower threshold [5,6]. ^4^ ESMO and SEOM suggest >40,000–50,000/μL [1,2]. VTE: venous thromboembolism; LMWH: low-molecular-weight heparin.

**Table 3:** Platelet count thresholds as contraindications to anticoagulation (adapted from Leader et al. [59]).

Group	Variables	Anticoagulation ICH Risk ^1^
Cancer type	High-grade glioma	Intermediate
	Melanoma, renal cell carcinoma, thyroid cancer, choriocarcinoma	Uncertain
Brain tumor location	Intraventricular or convexity location in meningiomas	Uncertain
Prior ICH	Prior intratumoral hemorrhage on imaging	Uncertain
	Larger ICH (≥10 mL volume)	Higher
Platelet count	<50,000/μL	Higher
	<100,000/μL	Intermediate
Antiplatelet therapy	Combined with anticoagulation ^2^	Intermediate
VEGF inhibitors ^3^		Uncertain
Chronic kidney disease ^4^		Uncertain
Stereotactic radiosurgery ^5^		Uncertain

^1^ Additive ICH risk with anticoagulation. ^2^ Antiplatelet therapy alone does not appear to increase risk of ICH in brain tumors. ^3^ Potential risk of ICH in glioma but less evidence with brain metastases. ^4^ Established risk factor for anticoagulation hemorrhage with some evidence in patients with brain metastases on anticoagulation. ^5^ Potential ICH risk factor with and without anticoagulation in small cohorts of patients with melanoma brain metastases. ICH: intracranial hemorrhage; VEGF: vascular endothelial growth factor.

## Data Availability

No new data were created or analyzed in this study. Data sharing is not applicable to this article.

## Abbreviations

The following abbreviations are used in this manuscript:

CAT	Cancer-associated thrombosis
VTE	Venous thromboembolism
DVT	Deep vein thrombosis
PE	Pulmonary embolism
CNS	Central nervous system
CVC	Central venous catheter
SEOM	Spanish Society of Medical Oncology
ESMO	European Society for Medical Oncology
ASCO	American Society of Clinical Oncology
NCCN	National Comprehensive Cancer Network
ITAC	International Initiative on Thrombosis and Cancer
SEMI	Spanish Society of Internal Medicine
SETH	Spanish Society of Thrombosis and Hemostasis
RCT	Randomized controlled trial
VKA	Vitamin K antagonists
aPTT	Activated partial thromboplastin time
SmPC	Summary of product characteristics
UFH	Unfractionated heparin
LMWH	Low-molecular-weight heparin
HIT	Heparin-induced thrombocytopenia
DOAC	Direct oral anticoagulant
IVCF	Inferior vena cava filter
CVC-AT	Central venous catheter-associated thrombosis
PICC	Peripherally inserted central catheter
ESC	European Society of Cardiology
ESVS	European Society for Vascular Surgery
CHEST	American College of Chest Physicians
BSH	British Society for Haematology
SINuC	Società Italiana di Nutrizione Clinica e Metabolismo
ESPEN	European Society for Clinical Nutrition and Metabolism
ASH	American Society of Hematology
ISTH	International Society on Thrombosis and Haemostasis
SOR	French National Federation of Cancer Centers
AAGBI	Association of Anaesthetists of Great Britain and Ireland
AAS	Scandinavian Society of Anaesthesiology and Intensive Care Medicine
UEDVT	Upper extremity deep vein thrombosis
SPVT	Splanchnic venous thrombosis
PHT	Portal hypertension
TIPS	Transjugular intrahepatic portosystemic shunt
ICH	Intracranial hemorrhage
CT	Computed tomography
MRI	Magnetic resonance imaging
CTCAE	Common Terminology Criteria for Adverse Events
CrCl	Creatinine clearance
SC	Subcutaneously
ULN	Upper limit of normality
BMI	Body mass index
